# *IDH1^R132H^* Mutation Increases U87 Glioma Cell Sensitivity to Radiation Therapy in Hypoxia

**DOI:** 10.1155/2014/198697

**Published:** 2014-05-07

**Authors:** Xiao-Wei Wang, Marianne Labussière, Samuel Valable, Elodie A. Pérès, Jean-Sébastien Guillamo, Myriam Bernaudin, Marc Sanson

**Affiliations:** ^1^Université Pierre et Marie Curie-Paris 6, Centre de Recherche de l'Institut du Cerveau et de la Moëlle épinière (CRICM), UMR-S975, 75013 Paris, France; ^2^INSERM, U 975, 75013 Paris, France; ^3^CNRS, UMR 7225, 75013 Paris, France; ^4^CNRS, UMR 6301 ISTCT, CERVOxy group, GIP CYCERON, Boulevard Henri Becquerel, BP 5229, 14074 Caen cedex, France; ^5^Université de Caen Basse-Normandie, UMR 6301 ISTCT, 14000 Caen, France; ^6^CEA, DSV/I2BM, UMR 6301 ISTCT, 14000 Caen, France; ^7^CHU de Caen, Service de Neurologie, Boulevard Côte de Nacre, 14000 Caen, France; ^8^AP-HP, Groupe Hospitalier Pitié-Salpêtrière, Service de Neurologie 2, 75013 Paris, France; ^9^Service de Neurologie 2, Groupe Hospitalier Pitié-Salpêtrière, 75651 Paris Cedex 13, France

## Abstract

*Objective*. *IDH1* codon 132 mutation (mostly Arg132His) is frequently found in gliomas and is associated with longer survival. However, it is still unclear whether *IDH1* mutation renders the cell more vulnerable to current treatment, radio- and chemotherapy. *Materials and Methods*. We transduced U87 with wild type *IDH1* or *IDH*1^*R*132*H*^ expressing lentivirus and analyzed the radiosensitivity (dose ranging 0 to 10 Gy) under normoxia (20% O_2_) and moderate hypoxia (1% O_2_). *Results*. We observed that *IDH*1^*R*132*H*^ U87 cells grow faster in hypoxia and were more sensitive to radiotherapy (in terms of cell mortality and colony formation assay) compared to nontransduced U87 and *IDH1* ^*wt*^ cells. This effect was not observed in normoxia. *Conclusion*. These data suggest that *IDH*1^*R*132*H*^ mutation increases radiosensitivity in mild hypoxic conditions.

## 1. Introduction

The* IDH1* gene encoding the cytoplasmic NADP+-dependent isocitrate dehydrogenase—and more rarely* IDH2*, encoding the mitochondrial isoform—are frequently mutated in gliomas, especially low grade gliomas and secondary glioblastomas [1].* IDH1/IDH2* mutation is associated with better clinical outcome, whatever the grade, but it is still not clear whether it is merely a prognostic marker or a predictor of the response to radiotherapy or chemotherapy [2–6]. Recent data* IDH1/IDH2* mutation results in a new enzyme function catalyzing the NADPH-dependent reduction of alpha-ketoglutarate to D-2-hydroxyglutarate (D-2HG) [7].* IDH1/IDH2* mutations result in D-2HG accumulation and lowering NADPH levels. On one hand D-2HG inhibits various alpha-ketoglutarate dependant reactions, including histone and DNA demethylation, and is likely to promote—rather than inhibit—HIF1*α* degradation [8–11]. On the other hand, low NADPH levels might sensitize tumors to oxidative stress, potentiating response to radiotherapy, and may account for the prolonged survival of patients harboring the mutations.

Since the majority of gliomas are poorly responsive to current treatment regimens, the ability to enhance cell radio-chemosensitivity would be of clinical benefit. In this study, we characterized the impact of* IDH1* mutation on U87 glioma cell growth and radiosensitivity.

## 2. Methods and Materials

### 2.1. Cell Culture and Hypoxia Treatment

The human glioblastoma cell line U87 MG (HTB14) was obtained from the American Type Culture Collection (ATCC, Rockville, MD) and maintained in Dulbecco's modified Eagle's medium (DMEM), supplemented with 10% fetal bovine serum (FBS) and 1% penicillin/streptomycin. Normoxic cells (21% O_2_) were grown in a humidified-air atmosphere incubator containing 95% air/5% CO_2_ at 37°C. Hypoxia experiments were performed in a controlled atmosphere chamber (INVIVO2 1000, Ruskinn, Awel, France) set at 1% O_2_, 94% N_2_, and 5% CO_2_ at 37°C.

### 2.2. Production of Recombinant Expression Lentiviruses

A recombinant pLenti7.3/V5-TOPO expression vector (Invitrogen's ViraPowerTM HiPerformTM Lentiviral Expression Systems; catalog number K5320-00) containing the human* IDH1* wild type and* IDH1* ^*R132H*^ cDNA was generated. The expression clones and the ViraPower Packaging Mix were cotransfected into the 293FT Cell line to produce lentiviral stocks, which were used to transduce the mammalian U87 cell line. U87-*IDH1* ^*wt*^ and U87-*IDH1* ^*R132*^ stable cell lines were acquired using EmGFP selection by flow cytometry. The constructs was verified by DNA sequencing and RT-qPCR analysis.

### 2.3. Cell Proliferation Assay in Normoxia and in Hypoxia

To evaluate the impact of* IDH1* mutation on cell growth in normoxia and in hypoxia by trypan blue dye exclusion method, U87, U87-*IDH1* ^*wt*^, and U87-*IDH1* ^*R132H*^ cells (4000/well) plated in 24-well plates (6 plates in total) were incubated at 37°C for six hours in normoxia to adhere; then 3 plates were removed at 37°C in the controlled atmosphere chamber overnight. At 1, 3, and 7 days after exposure to normoxia and hypoxia, the cells were trypsinized, and the number of viable cells per well was determined by counting with trypan blue. The experiment was performed three times in triplicate each.

### 2.4. Comparative Cell Viability Assay after Irradiation, in Normoxia and in Hypoxia

To evaluate the effect of* IDH1* ^*R132H*^ in the response to radiotherapy, U87 cells,* IDH1* ^*wt*^-U87, and* IDH1* ^*R132H*^-U87 cells were plated (4.103 per well) in 24-well plates. Six hours later at 37°C in normoxia, plates were either kept in normoxia or incubated in the controlled atmosphere chamber 1% O_2_ overnight. The next day, cells were irradiated with doses ranging from 0 to 10 Gy in order to determine the most discriminating dose. Cells were fixed in paraformaldehyde (PFA) 4%, then stained with Hoechst 33342 (10 *μ*g/mL in PBS, Sigma-Aldrich, France) and photographed in a blinded fashion under fluorescence (4 wells per condition; 4 photographs per well) at 24 h, 48 h, and 120 h, respectively. Cells were counted with ImageJ software (Rasband, WS, ImageJ, US NIH).

### 2.5. Colony-Formation Assay in Normoxia and in Hypoxia

U87,* IDH1* ^*wt*^-U87, and* IDH1* ^*R132H*^-U87 cells were plated in 6-well containing 0.3% base agar layer. Six hours later, cells were either incubated in the hypoxic or normoxic chamber overnight. The next day, the cells were treated by radiotherapy at the Radiotherapy Department of the Centre de Lutte Contre le Cancer (CLCC) François Baclesse (Caen, France) using an X-ray generator with doses ranging 0–8 Gy (Therac 15-Saturne with a dose rate of 2 Gy/min) and then incubated again for colony formation. One month later, the colonies were fixed in 20% ethanol and stained with 0.05% crystal violet. Colonies that contained more than 50 cells were counted. Survival was calculated as the average number of colonies counted divided by the number of cells plated multiplicated by plating efficiency (PE), where PE is the fraction of colonies counted divided by cells plated without radiation. The clonogenic survival data were generated using JMP software. The experiment was performed five times in triplicate each.

### 2.6. Statistical Analysis

Results obtained* in vitro* were expressed as mean ± SEM Image analysis was performed with in-house macros under the ImageJ Software (Rasband, WS, ImageJ, US NIH). All statistical analyses were determined using post hoc tests after significant ANOVA. Values of *P* < 0.05 were considered statistically significant.

## 3. Results

### 3.1. Transduced Cells Express High Quantities of* IDH1* ^*wt*^ and* IDH1* ^*R132H*^

**Figure 1:**
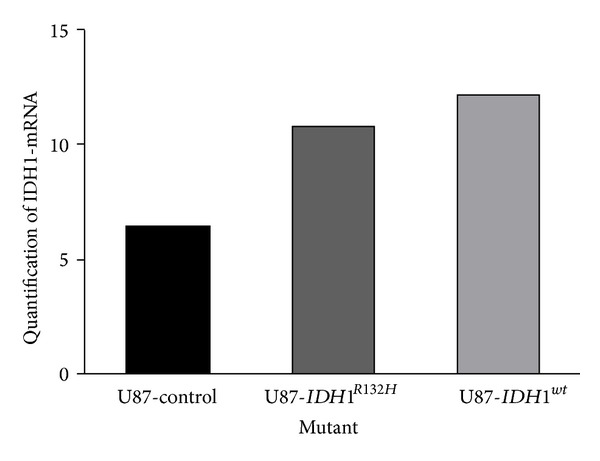
Real time PCR quantified the expression of the* IDH1* ^*wt*^ and* IDH1* ^*R132H*^ transduced genes.

The presence of* IDH1* ^*R132H*^ transduced gene was confirmed by DNA sequencing. Real time PCR showed a high expression of gene* IDH1* ^*wt*^ and* IDH1* ^*R132H*^ in transduced U87 cells compared to nontransduced cells (Figure 1).

### 3.2. *IDH1* ^*R132H*^ Expressing U87 Glioma Cells Grow Faster in Hypoxia

**Figure 2:**
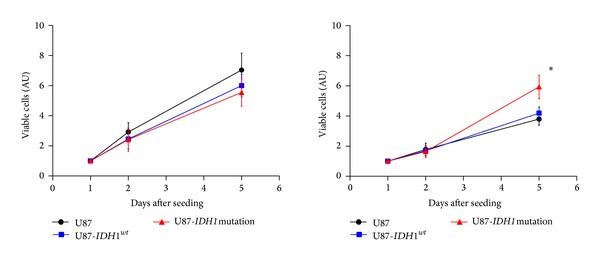
Effect of* IDH1* ^*R132H*^ on U87 cell proliferation. U87,* IDH1* ^*wt*^-U87, and* IDH1* ^*R132H*^-U87 cells were incubated in normoxia 20% (left) or hypoxia 1% (right) and cells were counted after 1, 3, and 7 days.

We determined whether* IDH1* ^*R132H*^ expression directly influences cell growth in normoxia and in hypoxia. The viable cell number per well was determined by counting with trypan blue at 1, 3, and 7 days after incubation in normoxia and in hypoxia. Proliferation rate of U87-*IDH1* ^*R132H*^ cells was significantly higher in normoxia than in hypoxia for all the three cell lines. In normoxia, U87, U87-*IDH1* ^*wt*^, and U87-*IDH1* ^*R132H*^ cells grew at the same rate, whereas U87-*IDH1* ^*R132H*^ grew faster than U87 and U87-*IDH1* ^*wt*^ in hypoxia (Figure 2).

### 3.3. Effect of Transduced* IDH1* ^*R132H*^ on Cell Viability upon Exposure to Doses Ranging 0 to 10 Gy in Normoxia and in Hypoxia

**Figure 3:**
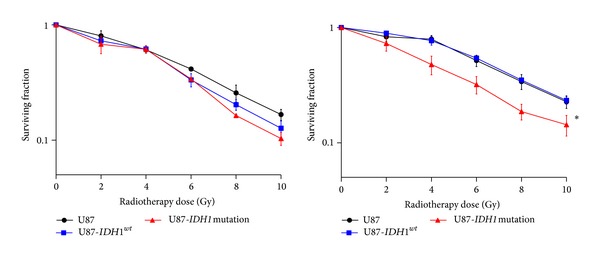
Effect of* IDH1* ^*R132H*^ on U87 cell viability after irradiation. Transduced cells were plated and then irradiated with doses ranging from 0 to 10 Gy, in normoxia (20%) (left) and in hypoxia (1%) (right). Cells were counted 5 days later.

To evaluate the role of* IDH1* ^*R132H*^ in the response to radiotherapy, U87, U87-*IDH1* ^*wt*^, and U87-*IDH1* ^*R132H*^ were exposed to different doses (range: 0–10 Gy): in normoxia the three cell lines showed the same radiosensitivity profile, whereas in hypoxia, the viability of U87-*IDH1* ^*R132H*^ cells was significantly lower after 5 days compared to control cells and* IDH1* ^*wt*^ cells (Figure 3) (13% versus 23% and 22% for a dose of 10 Gy, *P* < 0.001), respectively. This result suggests that* IDH1* ^*R132H*^ makes the cells more radiosensitive in hypoxic, but not in normoxic conditions.

### 3.4. Effect of Transduced* IDH1* ^*R132H*^ on Cell Mortality over Time following 8 Gy Irradiation in Normoxia and in Hypoxia

**Figure 4:**
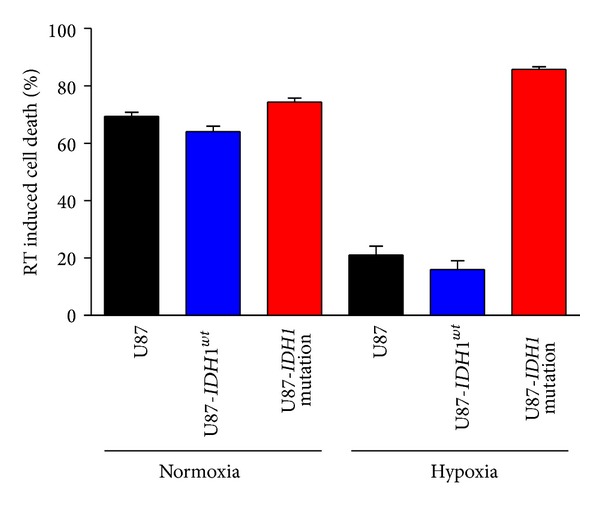
Cell viability after 8 Gy irradiation. Cells were counted before 8 Gy irradiation and 5 days after, in normoxia (20% O_2_) (left) and in hypoxia (1% O_2_) (right).

We quantified then cell death at 24 h, 48 h, and 120 h after 8 Gy irradiation. There was no substantial cell death after 24 h. The effect appeared at 48 h in both normoxia and in hypoxia (data not shown) and was maximal after 5 days. Cell death was significantly higher for* IDH1* ^*R132H*^ transduced cells in hypoxia but not in normoxia (Figure 4).

### 3.5. Radiosensitivity of U87-*IDH1* ^*R132H*^ in Hypoxia Is Confirmed by Colony-Formation Assay

**Figure 5:**
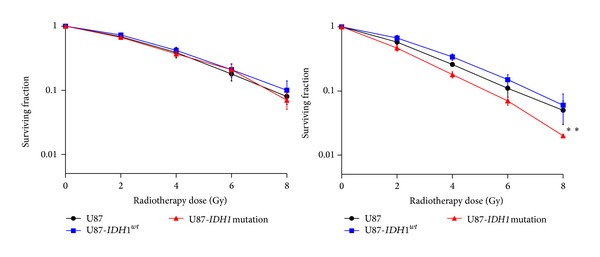
*IDH1* ^*R132H*^-U87 cells have a reduced colony forming cell ability after irradiation in hypoxia. U87,* IDH1* ^*wt*^-U87, and* IDH1* ^*R132H*^-U87 cells were plated 24 h before irradiation (0-2-4-6-8 Gy) in agar and incubated for one month in normoxia (20% O_2_) and in hypoxia (1% O_2_). The colonies were fixed in ethanol, stained with 0.05% crystal violet, and counted. Survival rate was estimated by the ratio between the colonies count and the number of cells plated, multiplicated by the plating efficiency.

A colony-formation assay was used to confirm the effect of* IDH1* ^*R132H*^ on the response to radiotherapy. Cells were treated with graded doses of radiation (0, 2, 4, 6, and 8 Gy). Colony-forming efficiency was determined 1 month later and surviving fractions were calculated. In normoxia, U87, U87-*IDH1* ^*wt*^, and U87-*IDH1* ^*R132H*^ had the same colony-formation capacity after radiotherapy. In hypoxia, the colony number of U87-*IDH1* ^*R132H*^ after radiotherapy was significantly lower than U87 and U87-*IDH1* ^*wt*^ (Figure 5). Thus, U87-*IDH1* ^*R132H*^ significantly sensitized U87 glioma cells to radiation.

## 4. Discussion

We observed here that* IDH1* mutated U87 grew faster in moderate hypoxic conditions (1% O_2_) than in normoxia (21% O_2_). This contrast with data obtained in normoxia,* IDH1* ^*R132H*^ overexpression in established glioma cell lines* in vitro*, resulted in a marked decrease in proliferation and mice injected with* IDH1* ^*R132H*^-U87 cells had prolonged survival compared to mice injected with* IDH1* ^*wt*^-U87 cells [12].

We found then that* IDH1* ^*R132H*^-U87 were more sensitive to radiotherapy in hypoxic condition. Indeed a high rate of cell proliferation is* per se* a sensitive factor of the radiation therapy response. But on the other hand,* IDH1/IDH2* mutated cells may be more sensitive to oxidative stress. The role of isocitrate dehydrogenase in cellular defense against oxidative stress has been suggested [13]. Indeed,* IDH1/IDH2* serves as a major source of cytosolic and mitochondrial NADPH production necessary to regenerate reduced glutathione (GSH) by glutathione reductase and for the activity of NADPH-dependent thioredoxin system, both are important in the protection of cells from oxidative damage [14, 15]. Thus, the decrease of NADPH in* IDH1/IDH2* mutated cells might result in an increase of ROS that can damage DNA. Partially in line with our results, U87 cells transduced with* IDH1* ^*R132H*^ or* IDH2* ^*R172K*^ demonstrated increased sensitivity to radiation but the effect observed in normoxia and hypoxic conditions was not investigated [16].

Despite hypoxia being considered as a factor of radioresistance, we observed here a radiosensitizing effect of* IDH1* ^*R132H*^ in glioblastoma cell line in hypoxic but not in normoxic condition. Until recently,* IDH1/2* mutations were believed to result in the stabilization of HIF1*α* [10, 17]. Interestingly Koivunen et al. [11] showed that D-2HG (but not L-2HG) instead of being an inhibitor of EGLN (HIF prolyl 4-hydroxylases) activity acts as a partial agonist of EGLN and promotes the degradation of HIF1*α*. Because HIF protects cells from irradiation therapy under hypoxic condition, we may hypothesize that* IDH* mutation, by inducing an inappropriate degradation of HIF, could make the mutated cell more vulnerable to RT.

In conclusion, this study suggests a radiosensitizing effect of* IDH1* ^*R132H*^ in glioblastoma cell lines U87 grown under mild hypoxic conditions, which are close to* in vivo* conditions. We need to confirm this finding on clinical setting: the 1p19q codeletion is a known marker of chemosensitivity. Whether the* IDH1/2* mutation is a marker of radiosensitivity should be determined. The ongoing EORTC trial on low grade gliomas, which randomizes radiotherapy versus chemotherapy in low grade gliomas at progression and includes also a prospective observational cohort, will be pivotal to answer this question.
